# Supporting meningitis diagnosis amongst infants and children through the use of fuzzy cognitive mapping

**DOI:** 10.1186/1472-6947-12-98

**Published:** 2012-09-04

**Authors:** Vijay K Mago, Ravinder Mehta, Ryan Woolrych, Elpiniki I Papageorgiou

**Affiliations:** 1 The Modelling of Complex Social Systems (MoCSSy) Program, The IRMACS Centre, Simon Fraser University, Burnaby, Canada; 2, Mehta Child Care Centre, Punjab, Sangrur, India; 3 Gerontology Research Centre, Simon Fraser University, Burnaby, Canada; 4 Department of Informatics and Computer Technology, Technological Educational Institute of Lamia, Lamia, Greece

## Abstract

**Background:**

Meningitis is characterized by an inflammation of the meninges, or the membranes surrounding the brain and spinal cord. Early diagnosis and treatment is crucial for a positive outcome, yet identifying meningitis is a complex process involving an array of signs and symptoms and multiple causal factors which require novel solutions to support clinical decision-making. In this work, we explore the potential of fuzzy cognitive map to assist in the modeling of meningitis, as a support tool for physicians in the accurate diagnosis and treatment of the condition.

**Methods:**

Fuzzy cognitive mapping (FCM) is a method for analysing and depicting human perception of a given system. FCM facilitates the development of a conceptual model which is not limited by exact values and measurements and thus is well suited to representing relatively unstructured knowledge and associations expressed in imprecise terms. A team of doctors (physicians), comprising four paediatricians, was formed to define the multifarious signs and symptoms associated with meningitis and to identify risk factors integral to its causality, as indicators used by clinicians to identify the presence or absence of meningitis in patients. The FCM model, consisting of 20 concept nodes, has been designed by the team of paediatricians in collaborative dialogue with the research team.

**Results:**

The paediatricians were supplied with a form containing various input parameters to be completed at the time of diagnosing meningitis among infants and children. The paediatricians provided information on a total of 56 patient cases amongst children whose age ranged from 2 months to 7 years. The physicians’ decision to diagnose meningitis was available for each individual case which was used as the outcome measure for evaluating the model. The FCM was trained using 40 cases with an accuracy of 95%, and later 16 test cases were used to analyze the accuracy and reliability of the model. The system produced the results with sensitivity of 83.3% and specificity of 80%.

**Conclusions:**

This work suggests that the application and development of a knowledge based system, using the formalization of FCMs for understanding the symptoms and causes of meningitis in children and infants, can provide a reliable front-end decision-making tool to better assist physicians.

## Background

Meningitis is defined as an inflammation of the membranes and cerebrospinal fluid that encases and bathes the brain and spinal cord. It is a serious disease which can be life-threatening and may result in permanent complications if not diagnosed and treated early. The pathogenic development of the disease suggests that meningitis can be broadly categorized into three main types [1]. *Bacterial meningitis*, which is rare, but more serious and can be life-threatening if not treated immediately. *Fungal meningitis* is typically diagnosed in patients with pre-existing conditions that have a weakened immune system, such as those living with lupus or HIV. *Viral meningitis* is caused by a virus (can be acute or chronic), is more common, but is far less serious and those who are diagnosed usually make a full recovery.

Symptoms of meningitis amongst children can appear very quickly or may take several days to make themselves known and include: fever; irritability; headache; photophobia (eye sensitivity to light); stiff neck; skin rashes; jaundice; inability to feed; high pitched cry; lethargy; seizures. Early diagnosis and timely interventions are the most effective ways for preventing negative outcomes associated with the disease.

Whilst meningitis cases affect all age demographics, the World Health Organisation has observed the highest rates of infection in young children [2]. For example, bacterial meningitis predominantly affects younger children and most cases of viral meningitis occur in children under the age of five years [3]. Epidemiological studies suggest rates of about two to ten cases per 10,000 live births with children particularly vulnerable to meningitis between the ages of 3 months and 3 years [4]. Fatality rates vary from as low as 2% for infants to 20 - 30% for neonates and adults. Since the mid-1980s, as a result of the protection offered by current vaccines and an increased understanding of the mechanisms of the disease [5], the median age at which bacterial meningitis is diagnosed has shifted from 15 months to 25 years. Geographically, meningitis epidemics have been experienced in various parts of the world, with research suggesting that climate might be a contributory risk factor in the spread of the disease [6].

In addition to the symptomatic development and epidemiological spread of the disease, there are other known risk factors associated with meningitis which include social, environmental and economic determinants. Although most cases are isolated, the disease can spread amongst people living in close social proximity, and outbreaks have occurred in those areas where there is a higher degree of social interaction or in areas experiencing overcrowding [7] which promotes exposure and transmission. The research indicates that meningitis in more prevalent in poorer areas then in affluent areas, suggesting that there is also a strong socio-economic component to the development of the disease [8]. Indeed the risk of invasive meningococcal disease (leading cause of bacterial meningitis) in children is strongly influenced by unfavorable socioeconomic conditions [9]. Increased levels of poverty are also linked to identified barriers in terms of geography, income, and socio-cultural differences. Research has found that presenting for treatment and early management of the disease is compounded by issues related to geography (access to medical facility), income (cost of healthcare), or cultural differences (attitudes towards illness and disease) which prevent lower socio-economic groups from receiving treatment, increasing the risk of adverse outcomes. Others have suggested that improvements in access to healthcare and earlier treatment are more likely to reduce the rate of mortality from meningitis [10].

Physicians are confronted with a broad range of symptoms and risk factors which they need to take into account when assessing a patient with possible meningitis, and when establishing the consequences of various treatment options. The ways in which these symptoms and risk factors inter-relate and how they are identified by healthcare professionals are integral to improving outcomes from the disease.

At a macro level, a number of studies have shown that the diagnosis and treatment management of meningitis is a complex and challenging problem for government and healthcare agencies requiring novel approaches to its management and intervention [11-14]. This has involved the application of modelling approaches for diagnosis and treatment. Public health experts working at the health protection agencies have developed a model to determine if suspected meningitis is bacterial or viral in origin. Clinical prediction rules have also been used to develop bacterial meningitis scores that classify patients according to risk of contraction [15]. Some diagnostic decision rules for management of children with meningeal signs have also been proposed to assist in timely diagnosis and decision-making [13,16]. Diagnostic scores have been constructed to predict disease outcomes and have been applied to successfully identify at-risk patients [17,18]. Based on literature studies, the symptoms, clinical features and microbiological (lab) examinations are the principal factors contributing to the accurate diagnosis and risk assessment of meningitis.

In this paper, we are proposing a modelling approach to understanding meningitis which focuses on capturing the various symptoms associated with the disease, incorporating specific risk factors such as socio-economic determinants as derived from expert knowledge provided by physicians. This work models the complex problem of meningitis diagnosis and severity assessment using Fuzzy cognitive mapping (FCM), which is an effective knowledge representation and modelling technique [19]. Through the proposed technique, the paper will develop and validate a simple tool to predict the likelihood of viral or bacterial meningitis in younger infants and children.

The main scope of this work is the construction of a knowledge based tool for modelling meningitis diagnosis for children living in semi-urban areas of India. The meningitis diagnostic procedure typically involves close interaction between the biologist, pathologist and the paediatrician and involves extracting and analyzing blood samples from the patient. The diagnosis of meningitis is more challenging within semi-urban areas of Indian cities given the lack of healthcare infrastructure, co-ordination between healthcare agencies and professionals and the shortage of qualified physicians which potentially delay identification of the disease. Moreover, the average costs of laboratory tests and potentially long hospital stays as a result, make treatment expensive and unaffordable for the majority of patients living within developing countries [20].

A decision-making tool to assist in the diagnosis of meningitis provides the potential for healthcare professionals to arrive at a decision sooner and alleviates the cost burden to the patient if laboratory tests and hospital stays are not required. No previous research has explored FCM methodology for assessing and diagnosing meningitis. The tool proposed in this research is designed to aid paediatricians who are responsible for clinical decision-making regarding the treatment of children with meningitis which involves: diagnosing the disease and its severity and making decisions regarding the most appropriate treatment.

This paper is structured into five sections. The section on Methods briefly describes the principal aspects of FCM formalization and describes the construction of a tool to support the diagnosis of meningitis. The Results describes the accuracy of the tool in predicting the diagnosis of meningitis. Finally the Discussion and Conclusions emerging from the study are presented.

## Methods

### Main aspects of fuzzy cognitive maps

Fuzzy Cognitive Map methodology is a symbolic representation for the description and modelling of a complex system. Through FCM the behavior of the complex system is described in terms of concepts, where each concept represents a state or a characteristic of the system which dynamically interact with each other. FCMs are described by Kosko [19] as signed, directed graphs for representing association and computational inference processing, exploiting a symbolic representation for the description and modelling of a system. Concepts are utilized to represent different aspects of the system, and to describe their behavior. The dynamics of the system are represented through the interaction between individual concepts. FCM structures can be used to represent both qualitative and quantitative data. The construction of an FCM requires the input of human experience and knowledge of the system under consideration to ensure that it is rooted in the experiences of domain experts and has real world applicability.

FCM is a method for capturing and depicting the human perception of a given system. The method produces a conceptual model which is not limited by exact values and measurements, and thus is well suited to represent relatively unstructured knowledge and associations expressed in imprecise forms. FCMs describe particular domains using nodes (variables, states, inputs, outputs) and signed fuzzy relationships between them. The ‘fuzzy’ part establishes degrees of association, represented as links between the nodes of these diagrams, also known as concepts and is a dynamic tool for representing cause-effect relationships and feedback mechanisms [19]. The advantages of FCM modelling, such as simplicity, adaptability and capability of approximating abstractive structures, provide the potential to model complex problems [21]. FCM has been employed across many different scientific fields as a tool for modelling complex problems [22-34] and in the domains of medicine for analyzing complex medical processes and supporting clinical decision-making [35-44]. Also, FCM has the potential to capture and represent both static and dynamic factors, allowing knowledge to be represented from various sources including qualitative and quantitative sources (as fuzzy values), defining the association between concepts and to establish forward reasoning (decision-making on the basis of symptoms and clinical measurements).

**Figure 1:**
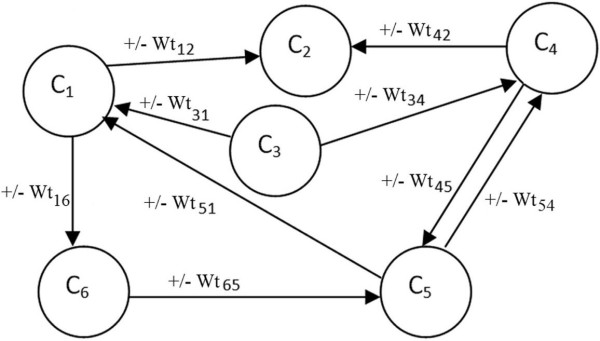
Basic structure of FCM.

Formally, an FCM consists of nodes-concepts, *C*_*i*_, *i* = 1,…, *N* where *N* is the total number of concepts. Each node-concept represents a key factor in the system, and is characterized by an activation value *A*_*i*_ ∈ [0,1], *i* = 1, …, *N*. This activation value allows the user to provide fuzzy input values to the inference algorithm. The concepts are interconnected through weighted arcs, which imply the relationships among them. A simple FCM with five nodes and nine weighted arcs is illustrated in Figure 1. Each interconnection between two concepts *C*_*i*_ and *C*_*j*_ have weight *W*_*ij*_, which is proportional to the strength of the relationship between *C*_*i*_ and *C*_*j*_. This is derived through the transformation of the fuzzy values assigned by the experts to numerical values. The sign of _*Wij*_ indicates whether the relationship between the two concepts is direct or inverse. The direction of association indicates whether the concept *C*_*i*_ is associated with the concept *C*_*j*_or vice versa. Thus, there are three types of weights:

(1) $$\left\{\begin{array}{c} W_{\text{ij}}>0\;\;\text{; expresses positive association}\\ W_{\text{ij}}=0\;\;\text{; expresses no association}\\ W_{\text{ij}}<0\;\;\text{; expresses negative association} \end{array}\right)$$

Human knowledge and experience of the system is used to determine the type and the number of nodes, in addition to the initial weights of the FCM. Having assigned values to the concepts and the weights, the FCM converges to a steady state. At each step, the value *A*_*i*_ of a concept is influenced by the values of concepts-nodes connected to it, and is updated according to the scheme [21]:

(2) $$A_{i}(k+1)=f\left(A_{i}(k)+\overset{N}{\underset{j\neq i,j=1}{\sum }}A_{j}(k)\times W_{\text{ji}}\right)$$

where *A*_*i*_ (*k*) is the value of concept *C*_*i*_ at step *k*, *A*_*j*_ (*k*) is the value of concept *C*_*j*_ at step *k*, *W*_*ji*_ is the weight of the interconnection from concept *C*_*j*_ to concept *C*_*i*_ and *f * is the threshold function that squeezes the result of the multiplication in the interval [0,1]. The transformation function is used to reduce an unbounded weighted sum to within a certain range, which is not robust for quantitative analysis, but allows for qualitative comparisons between concepts. The most commonly applied functions are continuous, although some research has utilized binary functions. A comparison of the different transformation functions for FCMs is provided by Tsadiras [45]. The sigmoid function *f * is selected for application within this paper as it is more suitable for diagnosis and planning:

(3) $$f(x)=1/(1+e^{-\mathrm{\lambda x}})$$

where *λ* > 0 is a parameter that determines its gradient in the area around zero. In the approach described here, diverse values of *λ* were examined through the validation analysis to establish the most feasible value. This function is selected since the values *A*_*i*_ of the concepts, by definition, must lie within [0,1] [46]. The interaction of the FCM results after a few iterations in a steady state, where the values of the concepts are not modified further. Desired values of the output decision concepts of the FCM support the operation of the simulated system.

### Development of FCM model for meningitis diagnosis support

Addressing meningitis is a complex process requiring an understanding of multifarious parameters, both symptoms and risk factors, to arrive at a decision regarding effective diagnosis and treatment. This paper is focussed on establishing the multiplicity of parameters together with the varying degrees of impact and dependency when diagnosing the presence or absence of meningitis among infants and children with a specific geographical focus on a semi-urban area of India, including barriers in terms of access, cost and efficiency of processing results. This all have potential implications in terms of diagnosing meningitis and bringing patients to treatment sooner.

**Figure 2:**
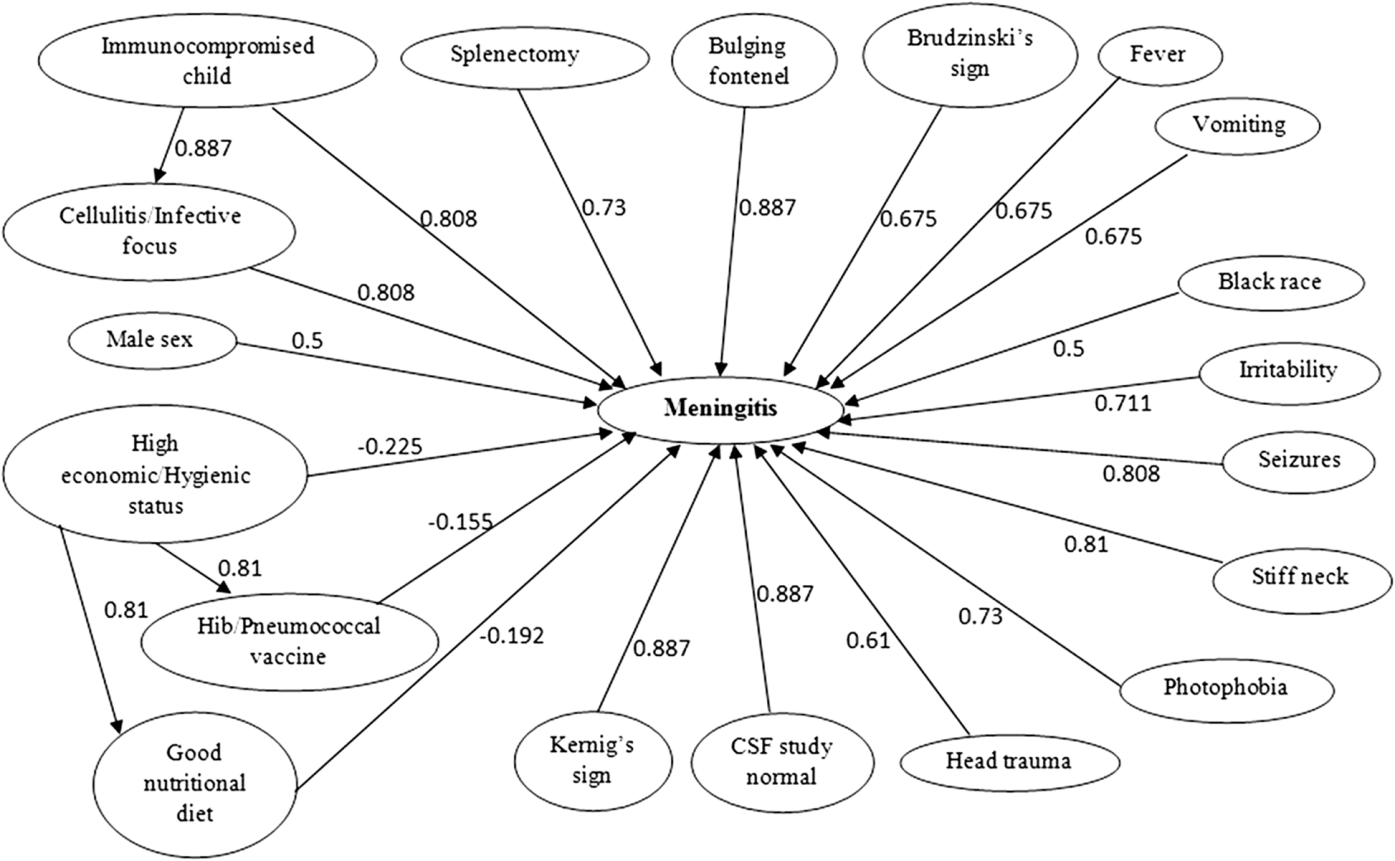
FCM Model for Meningitis Disease.

The development and design of an appropriate FCM for the description of a decision support system requires the contribution of human knowledge. In the research described in this paper, the expert knowledge comprised paediatricians who typically diagnose and treat meningitis within their everyday working practices. Paediatricians were collaboratively involved in the development of the FCM and in determining the operation and behavior of the system. In this study, a team of four paediatricians was formed to define the number and types of sign/symptoms and other risk factors used in determining the presence of the meningitis disease. The FCM model, consisting of 20 concept nodes (see Table 1), was designed by the research team after active dialogue and ongoing input from the paediatricians. Of the 20 concept nodes, 19 represent a list of the symptoms and risk factors considered by paediatricians in the diagnosis and treatment of meningitis and are illustrated in Figure 2. Whilst the figure includes some of the more established symptoms associated with meningitis, it also incorporates a risk factor associated with economic status. The opinions from paediatricians identified a link between lower levels of socio-economic status and lower levels of receiving the vaccine, which they use to establish the diagnosis of the disease and appropriate treatment. The central node ***Meningitis (Mn)*** is the basic decision concept which gathers the cause-effect interactions from all other input nodes.

**Table 1:** Concepts of the FCM model for diagnosing meningitis disease

**Concept node**	**Concept name**	**Concept node**	**Concept name**
C1	Sex (Male/Female)	C11	Seizures
C2	Cellulitis/infective focus	C12	Stiff neck
C3	Immunocompromised child	C13	Photophobia
C4	Splenectomy	C14	Head trauma
C5	Bulging fontanel	C15	CSF study abnormal
C6	Brudzinski’s sign	C16	Kernig sign
C7	Fever	C17	High economic/ hygienic status
C8	Vomiting	C18	Hib/Pneumococcal vaccine
C9	Black race	C19	Good nutritional status
C10	Irritability	C20	Possibility of Meningitis

**Figure 3:**
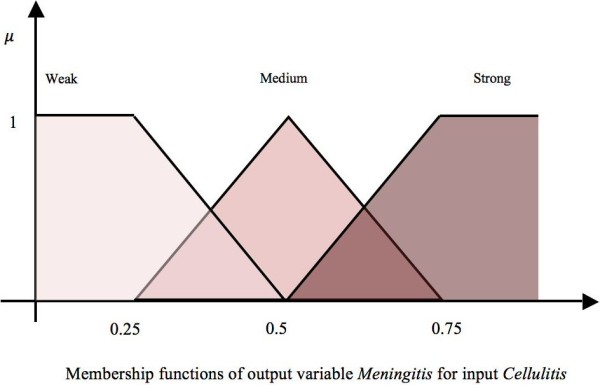
Three fuzzy sets for the concept “Cellulitis/Infective Focus”.

**Figure 4:**
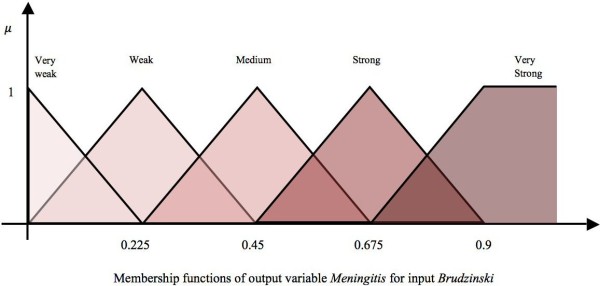
Five fuzzy sets for the concept “Brudzinski’s Sign”.

The 20 symptom/risk factor nodes represent those that the paediatrician will typically determine in their observations or discussions with the patient when diagnosing meningitis. Hence these nodes are considered observable nodes or input nodes. The impact of these nodes on *Mn* is determined using five or three fuzzy linguistic terms i.e., the association of these observable nodes on *Mn* can consist of three {*Weak*(*W*), *Medium*(*M*), *Strong* (*S*)} or five {*Very Weak* (*VW *), *Weak* (*W *), *Medium* (*M*), *Strong* (*S*), *Very Strong* (*VS*)} fuzzy sets and are shown respectively in Figure 3 and Figure 4. The fuzzy sets are equidistant and the parameters for the membership functions are defined intuitively by experts. This approach of relying upon expert opinion to define the fuzzy sets has been suggested in [47] and used in our previous research works [48,49]. Figure 3 shows the membership functions (triangular and trapezoidal) for output variable *Mn*, given the input variable *Cellulitis/Infective Focus*. Similarly, Figure 4 shows the membership functions for the output variable *Mn*, given the input variable *Brudzinski’s Sign*.

Each concept has a weighted impact on the decision node *Mn* and in some situations the node may have an impact on other observable nodes. For instance, the concept node representing the symptom ‘Immunocompromised child’ is having an impact on ‘Meningitis’ in addition to ‘Cellulitis/Infective Focus’. In the next subsection, we describe the method adopted to calculate the values on the FCM edges as these weights form the weight matrix used in Equation 1.

### Calculation of weights for FCM modelling meningitis

After defining fuzzy sets for the concept variables, each expert-paediatrician was asked to define the degree of influence between the FCM concepts and to describe their influence using an “if-then” rule. Following this, the four pediatricians were asked to infer a linguistic weight to describe the cause and effect relationship between each pair of concepts. Table 2 incorporates the paediatricians’ suggestions to describe the strength of the connection between concepts and the output as a numerical value to establish the weight of the association. This approach enables paediatricians to identify the weight of association between two concepts using readily understandable linguistic terms, and the FCM inference mechanism allows the various concepts to be interpreted in their entirety. Within this interpretation, there is no hierarchy among concepts, unlike that suggested by [50] and used by [51] to diagnose malaria.

**Table 2:** Strength of connections among concepts and the numerical weights produced by applying FL

**Edge**				**Feedback from Experts**				
**Start concept**	**End concept**	**Type of impact**	**Fuzzy sets**	**Expert 1**	**Expert 2**	**Expert 3**	**Expert 4**	**Defuzzified value**
Male Sex	Meningitis	Positive	W,M,S	M	M	M	M	0.5
Cellulitis ∖infective focus	Meningitis	Positive	W,M,S	S	S	S	S	0.808
Immuno-compromised child	Meningitis	Positive	W,M,S	S	S	S	S	0.808
Splenectomy	Meningitis	Positive	W,M,S	M	S	S	S	0.73
Bulging fontanel	Meningitis	Positive	VW,W, M,S,VS	VS	VS	VS	VS	0.887
Brudzinski’s sign	Meningitis	Positive	VW,W, M,S,VS	VS	S	VS	VS	0.81
Fever	Meningitis	Positive	VW,W, M,S,VS	S	S	M	M	0.562
Vomiting	Meningitis	Positive	VW,W, M,S,VS	S	S	M	M	0.562
Black race	Meningitis	Positive	W,M,S	M	S	S	M	0.658
Irritability	Meningitis	Positive	VW,W, M,S,VS	VS	S	S	S	0.711
High economic ∖hygienic status	Meningitis	Negative	VW,W, M,S,VS	W	W	W	W	-0.225
Hib ∖Pneumococcal vaccine	Meningitis	Negative	VW,W, M,S,VS	W	W	W	W	-0.155
Good nutritional status	Meningitis	Negative	VW,W, M,S,VS	VW	W	W	VW	-0.196
Kernig sign	Meningitis	Positive	VW,W, M,S,VS	VS	VS	VS	VS	0.887
CSF study abnormal	Meningitis	Positive	VW,W, M,S,VS	VS	VS	VS	VS	0.887
Head trauma	Meningitis	Positive	VW,W, M,S,VS	S	S	W	S	0.61
Photophobia	Meningitis	Positive	W, M,S	S	S	S	M	0.73
Stiff neck	Meningitis	Positive	VW,W, M,S,VS	S	VS	VS	VS	0.81
Seizures	Meningitis	Positive	W,M,S	S	S	S	S	0.808
Immuno-compromised child	Cellulitis ∖infective focus	Positive	VW,W, M,S,VS	VS	VS	VS	VS	0.887
High economic ∖Hygienic status	Hib ∖Pneumo- coccal vaccine	Positive	VW,W, M,S,VS	VS	VS	S	VS	0.81
High economic ∖hygienic status	Good nutritional status	Positive	VW,W, M,S,VS	VS	VS	VS	S	0.81

To illustrate how numerical values of weights are produced, an illustration of a sample edge is provided. This considers the strength of influence between the concept Brudzinski’s sign and the concept *Mn* (acts as the decision concept depicting the possibility of diagnosing ‘Meningitis’). The impact of concept Brudzinski’s sign on Meningitis could be: VW, W, M, S, or VS.

The relationships among the concepts have been defined in the form of IF-THEN rules of fuzzy logic. The opinions of four paediatricians were gathered in this context and are listed in columns 5-8 of Table 2. Expert-1 suggests that there is a Very Strong (positive) relationship between “Brudzinski’s sign” and “Meningitis”, while Expert-2 is of the opinion that there is a Strong (positive) relationship between these two concepts. Experts-3 and 4 agree with the opinion of Expert-1.

**1**^**st**^, **3**^**rd**^**and4**^**th**^**expert:**

*IF* (Brudzinski’s sign is ON) *THEN* the possibility to diagnose Meningitis is *VS*. Since this rule has been suggested by 3 experts out of 4, it is multiplied by 0.75.

**2**^**nd**^**Expert:**

*IF* (Brudzinski’s sign is ON) *THEN* the possibility to diagnose Meningitis is *S*. As this rule is approved by one expert, it is multiplied by 0.25.

**Figure 5:**
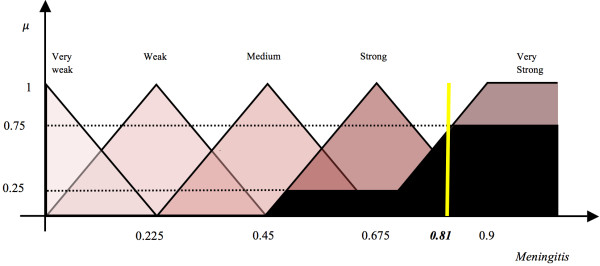
**Calculation of weight on the edge between concept *Brudzinski’s sign *and *Meningitis*.**

The crisp binary values of either *ON* or *OFF* allow the pediatricians to assume that if the condition given in the antecedent part of the rule is true, then there would be an implication on the consequent part of the rule. Using the **“max”** aggregation method, the **“centroid”** defuzzification method and the Mamdani inference mechanism, a crisp weight value (0.81) is calculated for the suggested relationship between these two concepts [21]. The procedure is shown in its entirety in Figure 5. The black portion of the fuzzy sets *Strong* and *Very Strong* is the outcome of the antecedent parts of the IF-THEN conditions. The *scaling* of these fuzzy sets is the result of a multiplication with weights assigned to these rules. This region is defuzzified using **“centroid”** method to produce a numeric value. A similar approach is employed to calculate numeric weights on the edges of the FCM model in order to form a weight matrix **W**. This weight matrix gathers the suggested weights of all interconnections among the concepts of the FCM model and is fixed throughout the experimentation. Currently, the values are shown in the last column of Table 2. Even though complex rules can be defined wherein the antecedent part of the rules may also be fuzzy but simple rules have been opted in order to determine the impact of the antecedent concept on the consequent concept as perceived by the domain experts. In the application of this system, the pediatrician can determine the patient’s symptoms by using the initial activation values of concepts using values in the range [0,1]. This implies that the input can be fuzzy in nature. Similarly, we opted to use a simple inference mechanism suggested by Mamdani, given that there cannot be more than four rules per edge, as represented in columns 5-8 of Table 2. An edge is represented by a row. This inference mechanism performs efficiently with a limited number of rules, otherwise the Sugeno algorithm [52] is a more suitable choice as the consequent part of the rules is presented by an equation as opposed to fuzzy sets.

### Source of data set

The paediatricians were supplied with a form containing the various input parameters which were to be labelled at the time of diagnosing meningitis among infants and children in their hospital settings. The paediatricians provided information from the cases of 56 different children patients, whose age ranged from 2 months to 7 years. The paediatricians’ decision was available for each of the cases as to whether they were treated for the meningitis disease or not. The paediatricians’ opinions were used as the “gold standard” for evaluating the model, as discussed in the section Results. The desired values for the system are the values derived from the opinion of the paediatricians. An ethics committee consisting of physicians and consultants at the Mehta Child Care Centre approved the study and the provision of the patient data.

The following section details the experiments conducted to refine the system by choosing the appropriate *λ* value and applying the model to a sample data set.

## Results

After the construction of the FCM tool, the system was refined to perform with higher levels of accuracy. This has been achieved by dividing the dataset into two parts: one for training the system to imitate real world decision-making and other for testing the system against clinical decisions regarding the diagnosis of meningitis. Forty cases, including twenty patients diagnosed with meningitis and twenty undiagnosed with meningitis, were used to train the system, and the remaining sixteen cases were used to establish the accuracy of the system. During training as well as testing, the information of patient cases were used to formulate the initial activation vector *A*, discussed in subsection Main Aspects of Fuzzy Cognitive Maps, which is then further processed using Equation 1. In other words, each patient state is represented as a vector of 19 concepts, where each concept is a sign or symptom or risk factor. Each one concept takes an initial value (activated value); if for example the Brudzinski sign is on, the concept takes the value 1, or in the case of “OFF”, it takes the value 0. The paediatrician can provide any value between 0 and 1. Then a concept vector is produced with the concept values and it is used in the FCM simulation algorithm (presented in [49]). A new (final) concept vector is produced after the system convergence (actually a system’s equilibrium point). The final value of the decision node *Mn* is the value which is presented in Additional file 1 and assessed for the system decision. The system converges to a different final state, if the initial patient conditions are different. An analytical description of how the values of FCM status change in relation to patient status is presented in previous works of Papageorgiou 2011 [53].

The efficacy of FCM reasoning is determined by the ability of the system to accurately determine the opinions of the paediatricians. The proposed system is used to predict the possibility of meningitis diagnosis in all training cases with different (*λ*) values of the threshold function defined in Equation 2. Authors of [54] suggest that an appropriate value for *λ*, used in sigmoid transformation function, be determined whilst training the system. Thus, during training phase, with different *λ* values, the system can achieve the optimal value for an efficient sigmoid threshold function used in FCM reasoning. For the system described here, the accuracy obtained for *λ* = {0.9,0.8,0.7,0.6,0.5,0.45,0.4,0.3,0.2,0.1} is reported in Additional file 1. The accuracy has been calculated using Equation 3.

(4) $$\begin{array}{c} \text{Accuracy}\;=\;\frac{\text{True}\;\text{Positive}\;+\;\text{True}\;\text{Negative}}{\text{True}\;\text{Positive}\;+\;\text{True}\;\text{Negative}\;+\;\text{False}\;\text{Positive}\;+\;\text{False}\;\text{Negative}} \end{array}$$

Five representative cases are reported in Table 3. Each row of the table includes the information for the patient, the decision taken by the pediatrician and the result produced by the system under various *λ* values. It was observed that the predictions made by the system were most accurate with the value of *λ* being 0.3.

**Table 3:** Five selected cases of forty examined patients during training phase

				**Result (in %) at λ**					
**Case Number**	**Age ∖Sex**	**Sign - symptoms**	**Decision by the Pediatrician**	**0.7**	**0.6**	**0.5**	**0.45**	**0.4**	**0.3**
1.	2 months ∖Female	Fever, Vomiting, Irritability, High Economic∖Hygenic Status, Hib∖Pneumococcal Vaccine,Good Nutritional Status,CSF Study Abnormal	No	68.5323	59.7309	49.9091	44.7308	39.4577	29.0711
2.	7 years ∖Male	Cellulitis ∖Infective Focus, Splenectomy, Brudzinski’s Sign, Fever, Vomiting, Irritability, Kernig Sign, CSF Study Abnormal, Stiff Neck	Yes	96.4422	93.2091	87.5451	83.4241	78.2209	82.7041
3.	3 years ∖Female	Immuno - compromised Child, Brudzinski’s Sign, Vomiting, Irritability, Kernig Sign, CSF Study Abnormal, Stiff Neck, Seizures	Yes	95.315	91.4263	84.9384	80.3924	74.8076	73.4831
4.	4 years ∖Male	Fever, High Economic ∖Hygienic Status, Hib∖Pneumococcal VaccineGood Nutritional Status	No	39.0668	32.3484	25.89	22.7909	13.8176	14.1353
5.	7 years ∖Female	Cellulitis ∖Infective Focus, Vomiting, High Economic∖Hygenic Status, Hib ∖Pneumococcal Vaccine,Good Nutritional Status,Head Trauma, Seizures	No	67.9496	59.1379	49.3509	44.2057	38.9751	28.6921

Cases 1 and 5 in Table 3 are the cases where a value of *λ* above 0.5 suggests that meningitis is present but in practice a treatment for meningitis disease is not recommended. This is supported by values of *λ* at 0.45, 0.4, and 0.3. The decisions of the paediatrician coincide with that of the system at *λ* = 0.3 for most of the examined patient cases i.e., 38/40.

It is imperative to understand the significance of selection of *λ* value. If *λ* value is close to 0, it converges very slowly but may produce results with high levels of accuracy; and if the value is near 1, it converges fast, but compromises accuracy. So, there is a trade-off between accuracy and the time of convergence which is dependent on the data set and the system under consideration. Our analysis shows that the system is performing accurately when *λ* = 0.3 and converges in approximately 20 iterations. The results of fine tuning are shown in Table 4.

**Table 4:** **Percentage of accuracy at various values of lambda-*λ ***

**Value of *λ***	**Accuracy of the system**
0.7	52.5%
0.6	60%
0.5	73.81%
0.45	83.33%
0.4	90%
0.3	95%
0.2	90%
0.1	50%

This experimentation implies that the transformation function has to be adjusted to produce high levels of accuracy. Authors of [54] suggested that “..sigmoid function can be considered an excellent decision support tool within any scope” and we have been able to fine tune this function by adjusting the *λ* value as per our data set.

After fine tuning our system, we conducted the experiment on the remaining data set which consists of 16 patient cases. Out of these, 6 were diagnosed and treated for meningitis disease by the pediatricians. The statistical information on the performance of our system is provided below:

(5) $$\begin{array}{lll} \text{Positive Predictive Value} & =\frac{\text{True}\;\text{Positive}}{\text{True}\;\text{Positive}+\text{False}\;\text{Positive}}\; & \;\\ & =\frac{5}{5+2}\;\\ & =71.4\%\; & \; \end{array}$$

(6) $$\begin{array}{cc} \text{Negative Predictive Value} & =\frac{\text{True}\;\text{Negative}}{\text{True}\;\text{Negative}+\text{False}\;\text{Negative}}\\ =\frac{8}{1+8}\\ =88.9\% \end{array}$$

(7) $$\begin{array}{lll} \text{Sensitivity} & =\frac{\text{True}\;\text{Positive}}{\text{True}\;\text{Positive}+\text{False}\;\text{Negative}}\; & \;\\ & =\frac{5}{5+1}\;\\ & =83.3\%\; & \; \end{array}$$

(8) $$\begin{array}{lll} \text{Specificity} & =\frac{\text{True}\;\text{negative}}{\text{False}\;\text{Positive}+\text{True}\;\text{Negative}}\; & \;\\ & =\frac{8}{2+8}\;\\ & =80\%\; & \; \end{array}$$

These results show that the system has a reasonably high level of accuracy and pediatricians can rely on the system in their clinical practices. The system is implemented in MATLAB R2012A, and provides a graphical user interface to the user which could be provided as a piece of fully functional software for pediatricians to use.

## Discussion

A key advantage of the FCM methodology used within this paper is that it can provide insight into the complexity of a specific problem through highlighting key concepts and feedbacks in the system which might otherwise remain unidentified. By underpinning the model development with the opinion of expert users, FCM is also capable of representing a system in a form that corresponds closely to the way humans perceive it. Therefore, the model is easily understandable, even by a non-technical audience, as each parameter can be easily interpreted within the context of the system as a whole. The FCM modeling technique also has an inherent flexibility and can be easily altered to incorporate new phenomenon, and if the behavior of the system operates differently than expected, FCM is more amenable to the modification of factors within the model.

The principal objective of the study described in this paper was to provide a decision making tool to the physicians working within infrastructural and economical constraints i.e., within a real world environment where solutions are sought to address external limitations that cause inefficiencies in the system. In this case the prohibitive costs to the patient of undergoing laboratory tests to detect meningitis and costly delays in the system which increase the likelihood of poorer outcomes. Even though the system has been able to achieve the desired functionality, there are a number of limitations of this modelling approach. Firstly, the experts who helped in designing the model assumed that the concept of high economic status is directly linked with achieving a good nutritional diet intake. This assumption is based on the prevalent socioeconomic conditions that the experts have witnessed within semi-urban India, which may vary over time and when applying the model across different geographical contexts [55]. Secondly, this study has incorporated 19 significant risk factors and signs/symptoms associated with meningitis associated in predicting and diagnosing meningitis, yet it is recognized that there are further factors which were not documented by experts but which are important in the diagnosis of the condition. Further work needs to be undertaken with experts-physicians in enhancing the proposed FCM model by adding more concepts and identifying the potential relationships among them, particularly across different socio-cultural contexts where risk factors and symptoms may vary. Thirdly, further work needs to be undertaken to clearly elucidate and comprehensively map the risk factors and symptoms associated with different forms of meningitis including baterial, viral and fungal strains of the disease. Lastly, the model described does not incorporate a temporal dimension, which is necessary to establish the urgency of treatment should the patient be diagnosed with meningitis i.e., urgent or non-urgent and should consider utilizing other modelling techniques.

## Conclusions

This study presents the results from research which sought to model expert users knowledge within a FCM decision support system which accurately diagnoses meningitis amongst infants and children. More specifically, this work proposes the application of a decision support tool based on the soft methodology of FCM to diagnose meningitis within specific individual cases. The developed software tool makes a decision based upon the state and condition of the patient as observed by the physician and therefore does not need access to specific patient data to determine the possibility of meningitis. The model was tested against a number of real patient cases demonstrating its capability as a dynamic decision making tool which could be used within clinical practice. Moreover, whilst the tool is designed to address cost and efficiency savings, it has the potential to be used not only within the clinical settings but as a mobile tool for application within more rural areas where access to healthcare facilities are more problematic. Further work will be undertaken by the authors to extend the FCM model described here to more clearly elucidate the breadth and depth of risk factors and symptoms associated with the disease amongst infants and young children, whilst applying the model across different geographical areas and increased numbers of patient cases to further validate the model.

## Competing interests

The authors declare that they have no competing interests.

## Author’s contributions

VKM and ELP developed the FCM model used in the diagnosis of meningitis among children and infants, and drafted the manuscript. RM provided the domain expertise and drafted the introductory section. RW assisted in manuscript development. All authors read and approved the final manuscript.

## Pre-publication history

The pre-publication history for this paper can be accessed here:

http://www.biomedcentral.com/1472-6947/12/98/prepub

## Supplementary Material

Additional file 1 **Appendix Table A.** FCM tool results in different percentage of accuracy for different *λ* values. Click here for file
