# Supplementary material for: Supporting meningitis diagnosis amongst infants and children through the use of fuzzy cognitive mapping
Source: BMC Med Inform Decis Mak. 2012 Sep 4;12:98. doi: 10.1186/1472-6947-12-98 (PMC3473237; doi:10.1186/1472-6947-12-98)
Supplement: Additional file 1 — Appendix Table A. FCM tool results in different percentage of accuracy for different λ values. [file 1472-6947-12-98-S1.pdf]

APPENDIX

Table A: FCM tool results in different percentage of accuracy for different λ values

		**Value of concept node Meningitis (*Mn*) after the system reaches an equilibrium state**										
#Patient	Treated for Meningitis	**λ=0.1**	**λ=0.2**	**λ=0.3**	**λ=0.4**	**λ=0.45**	**λ=0.5**	**λ=0.6**	**λ=0.7**	**λ=0.8**	**λ=0.9**	**λ=1**
1	Present/Yes	31.5462	57.382	75.2979	71.4849	77.2711	82.1007	89.2602	93.7929	98.9303	99.4419	99.7092
2	Present/Yes	31.0095	56.5784	74.5145	68.5349	77.3695	82.2576	89.8135	93.9915	96.6939	98.2401	99.0925
3	Absent/No	13.8945	20.8027	32.186	43.6734	49.2829	54.7068	64.7285	73.364	80.4396	85.9858	90.1764
4	Absent/No	18.8566	36.4183	40.2929	53.7139	59.9318	65.6952	75.628	83.3042	88.9373	92.8725	95.5187
5	Present/Yes	34.2039	61.243	78.916	74.7142	80.2604	84.7809	91.2551	95.1651	99.3346	99.6732	99.8397
6	Absent/No	6.8214	14.3181	22.4346	31.0449	35.4704	39.9323	48.7963	57.3298	65.23	72.2591	78.2895
7	Present/Yes	30.3209	55.5361	73.4831	74.8076	80.3924	84.9384	91.4263	95.315	98.6731	99.2883	99.6189
8	Absent/No	8.7664	18.3855	28.6921	38.9751	44.2057	49.3509	59.1379	67.9464	75.5079	81.7217	86.6417
9	Present/Yes	27.4986	51.1309	68.9368	66.0131	72.055	77.2894	85.4506	91.0099	97.8371	98.7643	99.2955
10	Absent/No	7.2143	15.2397	24.0044	33.3424	38.1397	42.963	52.4688	61.4481	69.5346	76.4829	82.2097
11	Present/Yes	28.2825	52.3755	70.2515	74.4313	72.2148	82.9911	89.9996	94.3513	98.1095	98.9387	99.4053
12	Absent/No	6.3625	13.3678	20.9786	29.0999	33.2999	37.5567	46.0937	54.4376	62.3005	69.4333	75.6768
13	Absent/No	4.2379	8.9492	14.1353	13.8176	22.7909	25.89	32.3484	39.0668	45.9059	52.6885	59.2323
14	Present/Yes	32.9262	59.4114	77.2299	73.1966	78.8643	83.5432	90.341	94.5443	99.163	99.5768	99.7862
15	Present/Yes	33.14	59.7211	77.5189	73.6411	79.2938	83.9438	90.6612	94.7785	99.1944	99.5946	99.7962
16	Absent/No	7.17	15.0385	22.534	32.5043	37.0922	41.6988	50.7823	59.4251	67.3189	74.2406	80.0907
17	Present/Yes	28.3101	52.419	70.2971	67.1651	73.1681	78.3317	86.2977	91.6456	98.1185	98.9444	99.4088
18	Absent/No	6.5697	13.7972	21.6371	29.9813	34.2848	38.6363	47.3263	55.7623	63.6489	70.7407	76.8916
19	Present/Yes	37.3858	65.6023	82.7041	78.2209	83.4241	87.5451	93.2091	96.4422	99.6284	99.8305	99.9227
20	Present/Yes	25.1628	47.3289	64.7772	57.4172	68.6532	74.0591	82.7494	88.9241	96.7854	98.0654	98.8388
21	Absent/No	15.3722	30.0346	34.5173	46.5754	52.9028	57.9238	67.9901	76.3994	83.1009	88.0219	91.946
22	Absent/No	14.8347	29.0306	42.0542	43.6736	49.2832	54.7073	64.7434	73.364	80.4397	85.9859	90.1759
23	Present/Yes	30.8331	56.3127	74.2532	71.0216	76.8913	81.803	89.1051	93.7306	98.7871	99.3569	99.6595
24	Absent/No	8.4318	17.6967	27.4625	37.6488	42.7586	47.8075	57.4865	66.3014	73.9667	80.3504	85.4723
25	Absent/No	14.2817	21.22	32.8001	44.4425	50.1063	55.5664	65.6047	74.1911	81.1723	86.6022	90.6736
26	Present/Yes	23.3511	44.2874	61.2992	59.6824	65.8064	71.3066	80.3614	87.0108	95.6511	97.2752	98.2981
27	Absent/No	16.3373	31.8251	36.0215	48.4212	54.861	59.9321	69.9611	78.2109	84.652	89.4633	92.93
28	Present/Yes	37.6049	65.8919	82.9443	78.6027	83.7781	87.867	93.4426	96.5997	99.6432	99.8381	99.9265
29	Absent/No	13.5119	20.3898	31.5769	42.9071	48.4603	53.8455	63.8446	72.5234	79.6893	85.3499	89.6596
30	Absent/No	20.2991	38.9915	54.942	61.2876	67.5815	73.1875	82.6702	88.7284	93.0788	95.8761	97.6082
31	Present/Yes	36.0795	63.8478	81.218	76.5039	81.9433	86.3144	92.4184	95.9683	99.5271	99.7776	99.8955
32	Absent/No	13.8945	20.8027	32.186	43.6734	49.2829	54.7068	64.7285	73.364	80.4396	85.9858	90.1764
33	Present/Yes	24.8018	46.7292	64.1018	61.9816	68.1008	73.5283	82.295	88.5649	96.5846	97.928	98.7464

34	Present/Yes	23.9695	45.3343	62.5111	60.6737	66.7989	72.2711	81.207	87.6954	96.0753	97.5746	98.5055
35	Present/Yes	28.3055	52.4118	70.2895	67.1586	73.1619	78.3259	88.4489	91.6422	98.117	98.9434	99.4082
36	Absent/No	8.8892	18.5654	29.0711	39.4577	44.7308	49.9091	59.7309	68.5323	76.0519	82.2016	87.0473
37	Present/Yes	35.456	62.993	80.4751	76.1398	81.5573	85.9235	92.0761	95.7104	99.4699	99.7471	99.8794
38	Present/Yes	35.3817	62.8903	80.385	76.6572	82.081	86.434	92.5017	96.0222	99.4627	99.0007	99.5253
39	Absent/No	20.6201	39.5582	48.4221	62.9014	69.1738	74.709	83.555	89.7203	93.7973	96.3701	97.9338
40	Absent/No	19.1554	36.9548	52.4009	52.7566	58.7652	64.3505	74.0458	81.7102	91.4049	94.0867	94.5187
	Wrong results	20	4	2	4	7	11	16	19	19	20	20
	Accuracy %	**50%**	**90%**	**95%**	**90%**	**82.50%**	**73%**	**60%**	**52.50%**	**52.50%**	**50%**	**50%**
